# Clinical presentation of autoimmune hepatitis in Pakistani children

**DOI:** 10.11604/pamj.2018.30.117.12692

**Published:** 2018-06-12

**Authors:** Ghous Bux Somroo, Ayesha Aslam Rai, Nasir Hassan Luck, Zaigham Abbas

**Affiliations:** 1 Department of Hepatogastroenterology, Sindh Institute of Urology and Transplantation, Karachi, Pakistani

**Keywords:** Autoimmune hepatitis, end stage liver disease liver disease, hypergammaglobulinemia

## Abstract

**Introduction:**

Autoimmune hepatitis (AIH) is one of the most common disorder resulting in end stage liver disease (ESLD) among children. Scarce data is available in this regard from Pakistan. In this study we have analyzed clinical and biochemical parameters of children suffering from this disorder.

**Methods:**

It was a cross sectional study conducted in the Department of Hepatogastroenterology, Sindh Institute of Urology and Transplantation (SIUT) Karachi from January 2005 to June 2016. Patients aged up-to 18 years were included. AIH was diagnosed by using International Autoimmune hepatitis group (IAIHG) pre-treatment and simplified AIH score. Patients with both probable and definite score were included. Biochemical, serological, sonographic and demographics were recorded at the time of diagnosis, liver biopsy was also performed in most of the cases. Data was analyzed by using SPSS ver.20 and p-value of < 0.05 was considered significant.

**Results:**

Total 51 patients were enrolled most of them were females (68.6%). Mean age of presentation was around 10 years. Males had statistically significant earlier age of presentation, p-value = 0.007. The most common presenting complain was jaundice. Hypergammaglobulinemia is seen in almost all patients. Type I AIH was the most common entity while Type II AIH was statistically more significant in males p-value = 0.019. Raised GGT was also seen in male patient specifically in Type II AIH, p-value = 0.001.

**Conclusion:**

Autoimmune hepatitis predominantly affects female children who have late age of presentation as compare to the males. Type I AIH was the most common while Type II AIH was more common in males and they also had raised GGT.

## Introduction

Autoimmune hepatitis (AIH) is a less common cause of End stage liver disease (ESLD) in Pakistani children as compared to viral, metabolic and hereditary causes. Multiple factors have been proposed to be involved in its pathogenesis including environmental and genetic predisposition [1]. It has predilection for females and can affect individual at any age [2] It usually presents with vague symptoms of fatigue, malaise, joint pain, abdominal pain, anorexia and nausea; furthermore, jaundice and hepatosplenomegaly can be found on examination [3]. Autoimmune hepatitis is classified in three types; Type I is the most common variety, almost 90% of patients have this variant of disease, it is associated with presence of HLA DR 3, 4 and 13 along with detection of antinuclear antibodies (ANA), anti-smooth muscle antibodies (ASMA) or anti soluble liver antigen/ liver pancreas (AntiSLA/LP) [4]. Type II is less commonly reported in around 10% of individuals, expressing anti liver kidney muscle antibodies (Anti LKM), anti-liver cytosol antibody (Anti LC-1) and associated with HLA DR 3 and 7, children usually have this type of AIH [4]. Type 3 is more or less similar to type I but may have a fulminant course. In this type Anti SLA/LP or anti Ro-52 are present. A criterion has been proposed by International Autoimmune hepatitis Group (IAIHG) for diagnosis of AIH [5]. Liver histology is of utmost importance in these cases, interface hepatitis is the hallmark of the disease while lympho-plasmacytic inflammatory infiltrate is a typical feature of AIH but the absence of these histopathological features does not exclude the diagnosis [6,7]. It has been reported that rosette formation, giant cell hepatitis, central necrosis, lobulitis with lymphoplasmacytic infiltrate and bile duct injuries on histopathology along with high serum level of Ig G are frequently observed in acute phase of AIH [8]. Limited data is available about disease spectrum of AIH in our region of the world, so the aim of this study was to assess the various clinical presentation of autoimmune hepatitis among Pakistani children.

## Methods

The study was conducted in the Hepatogastroenterology unit, Sindh Institute of Urology and Transplantation (SIUT) Karachi, and all patients under the age of 18 years, who were diagnosed and treated as AIH over the last eleven years from January 2005 to June 2016, were included prospectively in the study. Written informed consent was obtained from the parents or legal guardian. The study was approved by the ethical review committee of SIUT. All the patients falling under the criteria of probable AIH according to IAIHG and simplified criteria for the diagnosis of AIH were also included in the study [5, 9]. Patients were also screened for the presence of hepatitis B and C by using Enzyme linked immunosorbent assay (ELISA) and wherever appropriate serologies for hepatitis A (HAV), hepatitis E (HEV) and Human immunodeficiency virus (HIV) were also obtained, Wilson's Disease (WD) was also excluded on the basis of normal serum ceruloplasmin (>20 mg/dL) and absence of KF ring, and history was also taken in particular regarding use of any drug which can lead to drug induced liver injury (DILI) within past two weeks, were also excluded from the study. Blood samples was taken for liver function tests including alanine aminotransferase (ALT), aspartate aminotransferase (AST), gamma glutamyl transferase (GGT), serum globulins and immunoglobulin G levels (IgG). Autoimmune serological test were performed which included ANA, AMA, ASMA, Anti LKM and Anti SLA. HLA typing was also performed for 24 patients while liver biopsies were obtained from 45 patients under general anesthesia for children less than 14 years of age, while adolescents (aged 14 years or more) were given local anesthesia. All procedures were done by experienced radiologist under ultrasound guidance; biopsy specimens were stored in formalin bottles and sent for examination to the histopathology department at SIUT. Patient demographic characteristics like age, gender; laboratory investigation results and histopathology findings were compiled on predesigned proforma by the researcher. Diagnosis of AIH was made in the presence of raised gamma globulin levels of more than 1.1 times upper limit normal, presence of one or more auto antibodies ie: ANA, ASMA, Anti LKM or Anti SLA, by using IAIHG and simplified AIH score pre-treatment. Patient with both probable and definite AIH score were included in the study. Characteristic features on histopathology which included rosette formation, piecemeal necrosis, lobulitis, lymphoplasmacytic infiltrate or giant cell formation. Staging of fibrosis and grading of inflammation was done according to Batts and Ludwig scoring system and mofied Ishak scoring system [10,11]. Data was entered and analyzed in statistical software SPSS-20.0. Mean and standard deviation (SD) was calculated for continuous variables (age, BMI, duration of symptoms). Frequencies and percentages was evaluated for categorical variables. Stratification was done with respect to gender and types of AIH. Post stratification one way anova test was applied for types of AIH and simple T test and Chi square test was applied for gender. A p-value ≤ 0.05 was taken as significant.

## Results

Total number of patients included in the study were 51 out of which 35 (68.6%) were females (Table 1). The mean age of presentation is 10.1 ± 3.3 years (Table 2). Male had earlier age of presentation 9.5 ± 2.8 years and p-value was significant ( p = 0.007)(CI: 0.752-4.413) (Table 3)

**Table 1:** Basic demographic data, clinical and laboratory parameter of study population

Variables	Number (Percentage)
Gender	
Male	16 (31.4%)
Female	35 (68.6%)
Clinical Cirrhosis	
Present	35 (68.6%)
Absent	16 (31.4%)
History and General Physical Examination	
Hepatomegaly	6 (11.8%)
Splenomegaly	13 (25.5%)
Hepatosplenomegaly	25 (49%)
No visceromegaly	7 (13.7%)
Jaundice	44 (86.3%)
Abdominal pain	25 (49%)
Abdominal distension	32 (62.7%)
Edema	7 (13.7%)
Malaise	29 (56.9%)
Fever	16 (31.4%)
HLA Typing	
HLA DR3 and DR4	1 (2%)
HLA DR3	9 (17.6%)
Other HLA	5 (9.8%)
Not done	36 (70.6%)
Types of AIH	
Type I	34 (66.7%)
Type II	5 (9.8%)
Seronegative AIH	12 (23.5%)

**Table 2:** Clinical and biochemical parameters of study population (n=51)

Variables	Range	Mean±SD	Median
Age in years	4-17	10.16± 3.3	10
IAIHG score	10-21	13.88±2.73	14
AIH score	6-8	6.8±0.74	7
Hb (g/L)	5-13.7	9.63±1.82	9.7
Platelets (x 10^3^/µL)	22-375	140.53±80.48	126
TLC (x 10^3^/µL)	1.2-15.1	6.7±1.33	6.5
Urea (mg/dL)	4-51	19.06±9.97	17
Creatinine (mg/dL)	0.1-1.76	0.45±0.30	0.4
Albumin (g/dL)	1.3-4.4	2.8±0.73	2.9
T. Bilirubin (mg/dL)	0.12-49.41	4.13±7.67	1.7
D. Bilirubin (mg/dL)	0.012-25.78	2.07±4.23	0.59
Alkaline Phosphatase (U/L)	42-1039	345.60±229.12	287
ALT (U/L)	17-1510	136.14±220.53	78
AST (U/L)	24-844	149.2±171.43	78
GGT (U/L)	12-486	89.55±82.91	70
PT (in seconds)	10.7-36	15.37±4.98	14.1
INR	0.93-3.10	1.41±0.41	1.34
IgG (g/dL)	12.6-55.63	25.22±8.82	22.6
IgM (g/dL)	0.87-27.2	3.06±4.63	2.21
IgA (g/dL)	1.5-7.88	3.32±1.79	2.89
C3	0.3-3.53	0.97±0.67	0.98
C4	0.03-0.89	0.22±0.21	0.20

**Table 3:** Patient’s clinical, biochemical and histological parameters at time of diagnosis according to the gender distribution (n=51)

Parameters	Female ( n=35)	Male (n =16)	p-value
Age (mean ±SD)	12.1±3.4	9.5±2.8	0.007
Clinical Cirrhosis	24	11	1.00
ALT (mean ±SD) IU/L	137±251.6	134.1±136.4	0.966
D. Bilirubin (mean ±SD) mg/dL	2.1 ± 4.9	1.9±1.9	0.918
GGT(mean ±SD) IU/L	72.8±54.8	126±118.4	0.032
Albumin (mean ±SD) g/dL	2.8±0.7	2.6±0.6	0.19
INR (mean ±SD)	1.39±0.4	1.45±0.3	0.668
IgG (mean ±SD)	25.4±9.6	24.6±7.0	0.769
Plasma cell infiltrate	23	11	0.726
Rosette formation	10	6	1.000
Piecemeal necrosis	24	9	0.167
IAIHG	14.0±2.7	13.5±2.6	0.505
**Type of AIH**			
Type I	27	7	0.019
Type II	1	4	
Seronegative	7	5	

**Clinical features**: Among them 35 (68.6%) had evidence of clinical cirrhosis at the time of presentation. Jaundice was the most common presenting feature found in 44 (86.3%) followed by abdominal distension 32(62.7%) and abdominal pain 25(49%). However on clinical examination hepatosplenomegaly was found in 25(49%) patients as shown in Table 1.

**Types of AIH and score**: Type I AIH was diagnosed in 34(66.7%) of patients whereas Type II AIH with positive anti LKM Ab was found in 5(9.8%) of patients and seronegative AIH constitute 12(23.5%) of population in which no serologic marker was found to be positive. Out of 51 patients, 22(43%) had definitive diagnosis of AIH according to IAHIG score whereas 31(60.7%) had probable AIH according to the revised AIH revised scoring system. Mean IAHIG score was found comparable between both genders that is 14 ± 2.7 in females and 13.5 ± 2.6 in males and also according to the types of autoimmune hepatitis, p value 0.505. When characteristics of study population was compared according to the types of AIH by using one way anova test to determine statistically significant difference among different types of AIH to their clinical, biochemical and histological parameters. The results revealed statistically significant differences among the mean GGT levels F (41311) = 7.593, p = 0.001. Post-hoc Scheffe tests revealed statistically significant differences between GGT levels in three groups, type I (M = 69.1, SD = 50.7), type II (M = 205.6, SD = 159.5) and seronegative AIH (M = 99, SD = 82.7). It was also evident in this study that type II AIH was more common in males M:F (4:1) and had statistical significance, chi square test was applied (p = 0.019) (Table 3).

**Laboratory parameters**: Laboratory parameters at the time of presentation with mean value were summarised in table Table 1, Table 2. Mean value of gamma GT was significantly high in male patients126 ± 118.4 IU/L (p-value = 0.032)(CI= 4.660-101.569) (Table 3). Mean GGT was also found to be significant among various types of AIH (P value = 0.001) (Table 4) No significant differences were observed in complete blood picture, coagulation profile, liver enzymes, serum IgG levels, auto antibodies, gender and AIH type stratification and other lab parameters. Mean value ± S.D were given in Table 2. HLA typing was performed in 15 patients, 1 patient had both HLA DR3 and DR 4 alleles while 9 (17.6%) patients had HLA DR3 allele. (Table 1)

**Table 4:** Patient’s clinical, biochemical and histological parameters at time of diagnosis according to the types of AIH (n=51)

Parameters	Type I	Type II	Seronegative	p-value
Age (mean ±SD)	10.1±3.1	12.2±2.7	10±3.7	0.409
Gender (Male/Female)	7/27	4/1	5/7	0.019
Clinical Cirrhosis	21	4	10	0.325
ALT (mean ±SD) IU/L	133.1±255.2	80.4±45.0	167.8±149.5	0.758
D. Bilirubin (mean ±SD) mg/dL	1.9±4.6	1.4±1.2	2.8±4.0	0.762
GGT(mean ±SD) IU/L	69.1±50.7	205.6±159.5	99±82.7	0.001
Albumin (mean ±SD) g/dL	2.8±0.6	2.9±0.8	2.5±0.8	0.413
INR (mean ±SD)	1.3 ± 0.3	1.3±0.2	1.5±0.5	0.102
IgG (mean ±SD)	24.4±7.0	29.9±12.1	25.4±11.8	0.432
Plasma cell infiltrate	23	3	8	0.531
Rosette formation	14	0	2	0.035
Piecemeal necrosis	21	3	9	0.815

**Histological characteristics**: Liver biopsy was performed in 46(90%) patients. Typical histologic features observed were plasma cell infiltrates, rosette formation and piecemeal necrosis. These features were compared with types of AIH and among both genders. However comparison of histologic features according to the types of AIH revealed more typical features in Type 1 AIH as compared to type II and was found to be significant in rosette formation (p value 0.035) (Table 4) while rest of histological parameters had no statistical significance among two groups.

## Discussion

Autoimmune hepatitis is likely an immune mediated disease of liver, which has multifactorial pathogenesis but its exact etiology is yet to be known [3,12,13]. Mean age of presentation in this study was around 10 years which was similar to that reported by Karakoyun et al [14]. Another case series study done in Bahrain also has median age of 10 years [15]. In this study male has statistically significant earlier age of presentation as compare to the females. AIH is more common in females with Female: Male of 3.4:1 in this study. Similar findings had been reported in several other studies. The ratio of 4:1 was reported by an Argentinian study, Germans found 2.2:1 [16], Egyptians had proportion of 2.4:1 [17]. AIH in children has variable presentation. It can be asymptomatic or can present as acute liver failure. I this study around 68% of the children had the evidence of clinical cirrhosis at the time of their first presentation. A study done by Maggiore et al on seronegative AIH patients found that 3 quarters of children had an acute onset and more than 50% had signs of ALF [18]. Kogan et al found cirrhosis in around 47% of all symptomatic patients [19]. Jaundice was the most common presenting complain in 86% of the patients followed by abdominal distension and pain in this study. Another study conducted in same institute by Hassan et al reported fever as the most common presenting complain followed by jaundice and abdominal pain [20]. The diagnosis of AIH was made by using IAIHG score in the current study [5]. Mean IAIHG score was around 14 prior to treatment while post treatment follow up was not recorded in the current study and this pre-treatment score was almost similar to the Kogan et al [19]. IAHIG scores were comparable between both genders and all three types of AIH aswell. The serological investigations showed positive ANA in around 55% of the total patients. Type I was the most common followed by seronegative and type II AIH. The characteristics of three groups showed statistically significant difference among gender and GGT levels. Type II AIH was more common among males with raised GGT levels as compare to the other two types of AIH. The results of our study were in contrast to the Turkish population who had no statistical significance in the GGT levels of two groups [14].

Type I AIH was more common in our patients and similar trend had been observed by Karakoyun et al but they didn't find statistical difference in frequency among male and female [14]. HLA typing, a costly investigation was done in only 15 patients because of limited resources however most of the studied patients had HLA DR-3 allele. Hassan et al reported that HLA DR-3 is prevalent in normal population and reported HLA DR B1.13, HLA DR B1.14, HLA A2, HLA A9, HLA A10 and HLA A19 were more common in AIH population [20]. Other studied biochemical parameters and serum immunoglobulin levels had no statistical significance when compared according to the gender and types of AIH. Hypergammablobulinemia was seen in almost all of the patients, mean IgG levels were around 25.2 g/L which were comparable with Hassan et al [20]. Turkish children had statistically significant difference among the IgG levels of two genders but in this study although mean IgG was more in females but it had not attained the level of significance [14]. Liver biopsies were taken in 90% of the patients, staging and grading was done according to the Modified Ishak fibrosis score and Batts and Ludwig system [9,10]. The typical histological features of AIH includes dense mononuclear and plasma cell infiltration, interface hepatitis and rosette formation [3,6, 7, 21]. The most common histological finding in this study were lobulitis, plasma cell infiltration and piecemeal necrosis. Along with varying degree of fibrosis and inflammation bile ductular proliferation was noted in more than 50% of the patients. Similar findings has been observed by several other studies [20, 22-27]. In this study rosette formation had statistical significance in type I AIH. Moderate to severe inflammation was seen in more than 60% of the patients along with bile ductular proliferation in more than 50% of the patients. Similar findings were reported among symptomatic Israeli patients [19]. This study has used IAIHG and revised AIH score for the diagnosis of AIH and has also performed liver biopsy in majority of the patients which is considered as gold standard. It has highlighted the clinical and biochemical characteristics of these children at the time of presentation. This study has not collected the data about post treatment response but has tried to gather information about the trend of AIH among Pakistani children as scare data is available in this regard.

## Conclusion

Autoimmune hepatitis predominantly affects female children who have late age of presentation as compare to males. Type I AIH was the most common while Type II AIH is more common in males and they also have raised GGT.

### What is known about this topic

- Autoimmune hepatitis is more common in female;
- Had used validated scores for the diagnosis of autoimmune hepatitis.

### What this study adds

- Information about clinical spectrum of autoimmune hepatitis in children;
- Little information is available from this region of world about this disorder in children;
- It will help pediatric gastroenterologist in developing countries to know about the demographics and clinic presentation.

## Competing interests

The authors declare no competing interests.
